# Effects of Reducing Sugars on Colour, Amino Acids, and Volatile Flavour Compounds in Thermally Treated Minced Chicken Carcass Hydrolysate

**DOI:** 10.3390/foods13070991

**Published:** 2024-03-24

**Authors:** Xing Zhang, Shao-Quan Liu

**Affiliations:** 1 Department of Food Science and Technology, National University of Singapore, Science Drive 3, Singapore 117543, Singapore; e0669543@u.nus.edu; 2 National University of Singapore (Suzhou) Research Institute, 377 Lin Quan Street, Suzhou Industrial Park, Suzhou 215213, China

**Keywords:** chicken carcass, enzymatic hydrolysis, Maillard reaction, flavour compounds

## Abstract

This study investigated the changes in colour, amino acids, and volatile flavour compounds in the enzymatic hydrolysates of chicken carcasses containing different types and amounts of reducing sugars (xylose, arabinose, glucose, and fructose), so as to develop a chicken-based flavouring agent. Before heat treatment at 100 °C for 60 min, the chosen reducing sugars were separately added to the chicken carcass hydrolysate at its natural pH. Pentoses decreased pH more significantly than hexoses in the chicken carcass hydrolysate. The browning degree followed the pattern of pH decline, as pentoses caused more intense browning than hexoses, with xylose dosage having the greatest effect on the colour changes (ΔE). Fructose addition notably reduced free amino acids (FAAs) and cystine contents. Furthermore, phenylalanine decreased with increasing dosages of arabinose, xylose, and fructose. Glutamic acid content decreased significantly with fructose addition but showed insignificant changes with xylose. At the same dosage, the addition of pentoses resulted in the production of more sulphur-containing volatile compounds like methional, 2-[(methylthio) methyl] furan, and dimethyl disulphide than hexoses. Methional and furfural, which provide a roasted, savoury flavour, were produced by adding more xylose. Heat treatment with xylose also removed hexanal, the main off-odourant.

## 1. Introduction

A chicken carcass or frame refers to the remains of a chicken after it has been processed for its meat. It includes the skeletal structure and other parts of the chicken that are typically not consumed as meat. These carcasses can be used in various ways, such as making chicken broth or stock, pet food, or even in some industrial applications to extract valuable components [1]. Kampong chicken is the main chicken in Southeast Asia, which is used in dishes like Hainanese chicken rice. Free-range Kampong chicken is known for its toughness and unique flavour as well as its resistance to several diseases and high selling price [2,3]. Moreover, due to the lower growth rates, aged Kampong chickens have lower nutritional value and meat quality, making them difficult to market [4,5]. Thus, our study was focused on fresh Kampong chicken carcasses. Some studies have been conducted to assess the suitability of chicken by-products for the manufacture of value-added products such as protein hydrolysates and polyunsaturated fatty acids [6,7,8]. The efficient utilisation of by-products represents a comprehensive strategy for minimizing losses. Presently, various methods exist for maximizing the utility of chicken by-products, including their incorporation into animal feed, pet food, and soil enhancement; however, these avenues may entail considerable expenses. Moreover, the majority of these by-products are unsuitable for direct human consumption, underscoring the imperative to explore alternative means of enhancing their value. Additionally, inadequate reaction conditions in previous studies have led to the persistence of undesirable bitterness. Furthermore, a systematic comparison is lacking regarding the influence of pentoses and hexoses on the flavour of chicken carcass enzymatic hydrolysates during the Maillard reaction (MR).

Some peptides and amino acids from the enzymatically hydrolysed animal protein are recognised as taste-active substances and/or important flavour precursors [9]. For instance, some researchers developed a flavouring substance that could be added in instant noodle seasonings by the microencapsulation of mussel protein hydrolysates [9]. Previous research has indicated that most meaty flavour compounds have a similar structural unit to sulphur or oxygen heteroatoms on the adjacent two carbon atoms (one of the heteroatoms should be a sulphur atom) [10,11]. These distinctive structural units are further classified into six subclasses based on different bonding types, including 2-methyl-3-furanthiol (MFT) and 2-furfurylthiol (FFT), 1-alkylthio-2-butanethiols, 1-alkylthio-2-butanols, 2-methoxybutylalkyl sulphides, and 1-alkylthio-2-butanones [10].

Maillard reaction is one of the most important non-enzymic reactions in the food industry, which often occurs during the thermal processing of foodstuffs such as cooking meat, baking bread, and roasting coffee. Sun et al. (2014) suggested that the heat treatment of chicken bone hydrolysates was a promising way to prepare natural meat flavour enhancers [12]. The nature of the sugars and amino acids, as well as the pH, and the reaction temperature, time, and the water activity of the reaction system greatly affect the formation of volatiles and therefore influence the flavour profile of the final product [13]. The Maillard reaction products (MRPs) produced from chicken bone extracts may be exploited as possible natural flavourings in food applications [14]. Further, chicken bones were transformed into hydrolysates that could be nutritional and flavouring substances [15]. Recently, researchers demonstrated the effect of degree of thermal treatment on flavour generation from the Maillard reaction of xylose and chicken peptides; high temperature (≥100 °C) could remarkably increase the formation of meaty aroma, while lower temperature and longer heating duration tended to generate a broth-like taste (i.e., umami and kokumi) [16]. Furthermore, the taste enhancers that could lead to bitterness would be produced at a higher temperature (>120 °C); hence, relatively lower temperatures and longer heating time were needed [16]. This study aimed to further explore the valorisation of chicken carcass enzymatic hydrolysates, building upon our previous optimisation research [17]. To be specific, the objective was to promote the thermal generation of both volatile and non-volatile flavour compounds by incorporating four reducing sugars as flavour precursors (xylose, arabinose, glucose, and fructose). This approach was driven by several reasons: (1) in view of the lack of a reducing sugar in the chicken carcass hydrolysate, the supplementation of reducing sugars would be of great importance for both the colour and the volatile compound formation [18]; (2) previous research showed that pentoses tended to be more reactive than hexoses in the Maillard reaction [19], contributing to different volatile flavour compounds; (3) C-2 and C-3 OH groups are the key differences between xylose and arabinose, which could bring about the different stabilities in the hemiacetal ring that may cause different cleavages during heat treatment, resulting in diverse reactivity ratios and browning degrees [19]; and (4) the order of reactivity according to which aldoses are more reactive than ketoses; however, fructose (ketose) and glucose (aldose) are the counter examples; in other words, more reactive and more numerous reaction products can be generated from ketose (i.e., fructose) compared to aldose (i.e., glucose) [19]. The findings from the present study would be expected to help the poultry industry towards by-product valorisation by developing chicken-based flavouring substances from chicken carcasses. 

## 2. Materials and Methods

### 2.1. Preparation of Chicken Carcass Hydrolysates 

Fresh Kampong chicken carcasses were purchased from a local supermarket (Aw’s Market Fresh Anxin Kampong Chicken Bones, NTUC Fairprice Co Ltd., Singapore). The protease used was MEAP (mixed enzymes PB02 for animal proteolysis, Nanning Pangbo Biological Engineering Co., Ltd., Nanning, China), which is a protease/peptidase composed of fungal protease and peptidase derived from *A. oryzae*, endoprotease, and mixed exopeptidases, 200,000 units g^−1^, and the optimised hydrolysis conditions were described by Zhang et al. [17]. In general, prior to the experiment, 40 g of defrosted minced chicken carcasses was suspended in distilled water in a Duran bottle (Merck, Darmstadt, Germany) at a ratio of 1:1 (*w/v*). The content was manually mixed and then heated to 50 °C for 10 min in a water bath (SW22, Julabo, Seelbach, Germany). Then, a 4 h hydrolysis was immediately conducted, and the optimal parameters were determined to be a protease/substrate ratio of 3:100 (*w/w*), 51.20 °C, natural pH of 6.62 ± 0.05, and substrate/water ratio of 1:1 (*w/v*). The resulting slurry was filtered to eliminate the insoluble components using a coconut cloth filter to obtain a clear, transparent, light yellow-brown liquid. The highest DH for this hydrolysate was 45.44%. On a dry weight basis, the protein recovery was 50.45 ± 2.05%, including 7.29 ± 0.01% ash, 67.67 ± 1.55% crude protein, and 1.25 ± 0.04% fat. The majority of the peptides in this hydrolysate had smaller molecular weights (<5 kDa) [17].

### 2.2. Thermal Treatment of Chicken Carcass Hydrolysates 

A 40 mL volume of fresh chicken carcass hydrolysates was transferred into a 100 mL blue-capped Duran^®^ laboratory bottle (Merck, Darmstadt, Germany) with a specific amount of different reducing sugars (L-(+)-xylose, L-(+)-arabinose, D-(+)-glucose, and D-(-)-fructose, Merck, Darmstadt, Germany) as shown in Table 1 without any pH adjustments. The thermal treatment conditions were determined according to the previous research and preliminary test [16]. To be specific, all samples except the unheated control were heated at 100 °C for 1 h in a vertical autoclave (Hirayama MFG Corp., Kasukabe-ShiSaitama, Japan), to allow the formation of MRPs. At the end of autoclaving, the samples were cooled immediately in ice water. Unheated samples were prepared at room temperature (25 °C). At the end of heating, all samples were immediately cooled in an ice bath to prevent further reactions. 

### 2.3. Colour Analysis and pH Measurement

A reflectance spectrometer (CM-3500 d; Konica Minolta Sensing Inc., Osaka, Japan) connected to a D65 illumination with 10 observations was used to measure the colour. a* (red > 0, green < 0), b* (yellow > 0, blue < 0), and L* (lightness, black = 0, white = 100) were the three colour distribution parameters used in the measurement. The following equation was used to calculate the colour difference (∆E) using the L*, a*, and b* values obtained from unheated control samples [20].

(1) $$∆E=\sqrt{{\left(L_{t}-L_{0}\right)}^{2}+{\left(a_{t}-a_{0}\right)}^{2}+{\left(b_{t}-b_{0}\right)}^{2}}$$

The pH values of each sample were measured with a SevenCompact™ pH meter (Mettler-Toledo 135 International Inc., Columbus, OH, USA).

### 2.4. Determination of Sugars

The reducing sugar (xylose, arabinose, glucose, and fructose) contents were measured before and after heat treatment using a 1260 Infinity II preparative ultra-high performance liquid chromatography and reflective index detector (UHPLC-RID) system (Agilent, Santa Clara, CA, USA) with a modified protocol described by Chua et al. [21]. Before injection, 1.0 mL of each sample was filtered through a 0.2 μm Minisart RC 15 syringe filter (Sartorius, Goettingen, Germany), and then, 200 μL of each filtered sample was mixed with 200 μL of deionised (DI) water (arium^®^, Sartorius Stedim Biotech, Goettingen, Germany) and 600 μL of pure acetonitrile (Tedia, Fairfield, OH, USA) and kept at 4 °C for more than 2 h to allow protein precipitation. Each sample was centrifuged at 10,000× *g* at 4 °C for 15 min to remove the precipitate, and the collected supernatant was injected to the UHPLC system for sugar analysis. The sugars were separated using a Zorbax Eclipse Plus C18 column (150 × 4.6 mm, Agilent, Santa Clara, CA, USA) using a mobile phase containing 80% acetonitrile and 20% DI water at a flow rate of 1.4 mL/min at 40 °C. A series of external xylose, arabinose, glucose, and fructose standards were used for identification and quantification.

### 2.5. Analysis of Amino Acids

Amino acids were determined using the ARACUS Amino Acid Analyzer’s pre-set physiological separation algorithm (MembraPure, Berlin, Germany) following the procedure of Zhou et al. [22]. A lithium-cation exchange column was used along with ninhydrin (a reagent for post-column derivatisation) and eluents (Eluent A to Eluent F) provided by the manufacturer. Except for proline and hydroxyproline, which were identified at 440 nm, all other amino acids were detected at 570 nm. The amino acids were quantified using a calibration factor generated by MembraPure.

### 2.6. Analysis of Volatile Compounds

The analysis of volatile compounds in thermally treated chicken carcass hydrolysates was conducted using a modified protocol described by Vong and Liu [23]. The volatile compounds were extracted by headspace solid-phase microextraction (HS-SPME), coupled with gas chromatography–mass spectrometry and semi-quantified by flame ionisation detector (GC-MS/FID, Agilent 5975C, Santa Clara, CA, USA). An aliquot of 5 mL of samples was added to a 20 mL glass vial with a PTFE septum. An 85 mL carboxen/polydimethylsiloxane SPME fibre (CAR/PDMS, Supelco, Bellefonte, PA, USA) was used to extract the headspace volatile compounds at 60 °C for 30 min under 250 rpm agitation using a Combi PAL autosampler (CTC Analytics, Zwingen, Switzerland). The extracted compounds were separated on a DB-FFAP capillary column (60 m length, 0.25 mm i.d., 0.25 μm film thickness, Agilent, Santa Clara, CA, USA) with helium as the carrier gas at a flow rate of 1.2 mL/min. The conditions of separation were adopted from the study of Gao et al. [24]. Oven temperature was set at 40 °C for 3 min, then increased to 90 °C at a rate 5 °C per min, without holding, and increased to 230 °C at a rate 10 °C min per min with a holding time of 7 min. The identification of compounds was based on the comparison of their mass spectra with a database in NIST 17.0 and Wiley 275 MS libraries and their linear retention index (LRI) values. The LRI value of each compound was calculated using its retention time and a series of alkane standards determined in our laboratory. GC-FID peak areas were used to semi-quantify the volatiles, and the relative peak area (RPA) expressed in percentage was calculated in each major group of compounds.

### 2.7. Statistical Analysis 

All results were obtained from three independent experiments (conducted at different times). Two-way analysis of variance (ANOVA) and Duncan’s multiple range tests were performed for each data set by using SPSS^®^ 20.0 (SPSS Inc. Chicago, IL, USA). The bar graphs were plotted by GraphPad Prism 8.0. The FID peak areas were subjected to missing value estimation and normalisation by data scaling, that is, auto-scaling by mean-centring and dividing by the standard deviation of each variable prior to analysis.

## 3. Results and Discussion

### 3.1. pH Changes

**Figure 1:**
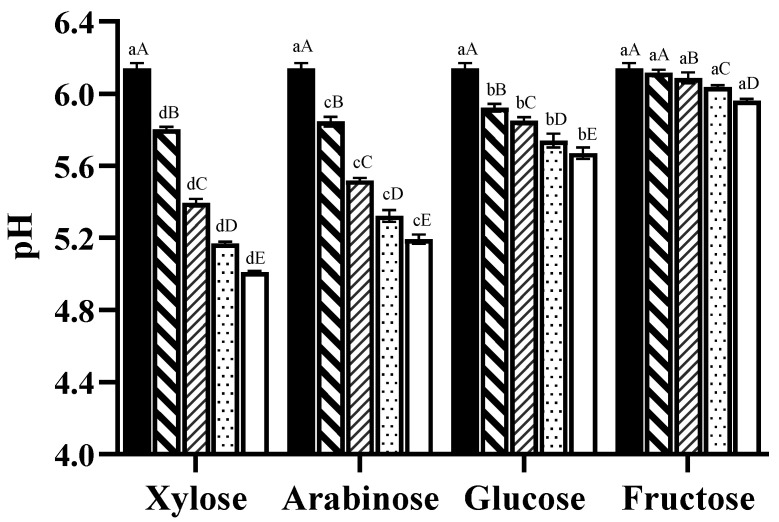
The changes in pH in chicken carcass hydrolysates with different added sugars (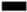 0%, 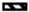 0.5%, 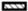 1.5%, 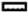 2.5%, 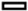 3.5%) during heat treatment at 100 °C for 1 h. ^a,b,c,d^ values within the same dosage on different types of sugars followed by the same letters are not significantly different (*p* > 0.05). ^A,B,C,D, E^ values between the different sugar dosages on the same sugar followed by the same letters are not significantly different (*p* > 0.05).

A general decreasing trend of pH was observed in all heated samples (from 6.14 to 5.01, 5.19, 5.63, and 5.97, respectively). Figure 1 shows an inverse correlation between the pH and the sugar dosage; pentoses showed a more significant pH reduction than hexoses. To be specific, the lowest pH of 5.01 ± 0.01 was found in the sample of xylose (Xyl-35), followed by arabinose (Ara-35) (5.19 ± 0.03), glucose (Glu-35) (5.67 ± 0.03), and fructose (Fru-35) (5.97 ± 0.01). The consumption of the amino groups and the accumulation of various organic acidic molecules such as formic acid and acetic acid during the Maillard reaction might be the major reason of pH decline [25]. Pentoses typically have greater impacts on decreasing pH compared to hexoses during the Maillard reaction for several reasons: some researchers revealed pentoses have less steric hindrance, so that the reaction with amino groups is promoted more than that with hexoses, and there is a greater probability for pentoses to be in their open-chain form because of the lower stability of the furanose ring (higher tension within the molecule, which forces the ring to open) compared to the pyranose ring. Pentoses may release more protons (H⁺ ions), causing the pH to drop more noticeably. Additionally, the disposition of OH groups affect the stability of the molecule (including the proportion of acyclic form), the initial steps of the Maillard reaction, and intermediate conversion, ultimately influencing global sugar reactivity [19,26].

The pH value affects the Maillard reaction significantly. It is known that when the pH is below seven, the Amadori rearrangement products (ARPs) undergo 1,2-enolisation to create 3-deoxyosone, which may then cyclise and dehydrate to produce 5-hydroxymethylfurfural (HMF) from hexoses and furfural from pentoses [27]. When the pH is greater than seven, ARPs undergo 2,3-enolisation to create 1-deoxyosone, which can subsequently break down into α-dicarbonyls (i.e., α-DCs) and reducing ketones [27]. In contrast, solutions with higher pH values exhibit a quicker reaction rate and a more intense colour development [28]. 

### 3.2. Colour Changes

The colour changes in controls and heated samples are shown in Figure S1 and Table 2. In general, ∆E values rose as each sugar dose increased. The relationship between the colour changes and the dosage of reducing sugar was evident. The browning degree shared the same trend as pH decline in that the pentoses had more intense browning than the hexoses. At 0.5%, fructose addition had deeper browning than glucose; however, all the fructose-added samples with higher dosages (1.5%, 2.5%, 3.5%) had overall colour changes (ΔE) opposite to those of the glucose samples, with the corresponding dosages being consistent with the previous research [29]. To be specific, the heated control showed the highest value of lightness (L*) and the lowest value of redness (a*), with a moderate yellowness (b*) level. After heat treatment with different types and dosages of reducing sugars, pentoses could result in lower L* and b* than hexoses. Pentoses-added samples, on the other hand, could also result in a relatively higher a*; among which, Xyl-15 sample had the lower L* (28.64 ± 0.52) and the highest a* (45.81 ± 0.18), with moderate b* (49.13 ± 0.95), indicating a dark brownish red and yellow colour. L* was mainly affected by the amount of browning pigments (i.e., melanoidins), while the amount of these pigments was influenced by the reactive degree of the Maillard reaction and caramelisation. Fructose has less accessible carbonyl function and a much lower energy open-chain form, which is characterised by a much lower reactive degree of Maillard reaction. Moreover, at the temperature used in the present study (100 °C, 1 h), caramelisation, which could have led to an overestimation of browning measurements in a fructose system, may have not occurred.

Besides the caramelisation of sugars, the Maillard reaction between reducing sugars and amino acids results in the production of melanoidins. Amadori rearrangement products (ARPs)/Heyns rearrangement products (Heyns) are crucial intermediates for the ensuing chain of events that generate volatile compounds and brown pigments (i.e., melanoidins), which directly affect the organoleptic qualities of foods [30]. Interestingly, previous research showed that cysteine was effective in preventing browning, but this phenomenon was only observed when cysteine was added to the system during the first mild reaction step [31,32], but the final reactions already happened in our study after 1 h heat treatment, which might be the reason why browning inhibition did not happen in the current study. The very low content of cysteine in the original chicken carcass hydrolysate may be another reason for the lack of browning inhibition [17].

### 3.3. Sugar Changes after Heat Treatment

**Figure 2:**
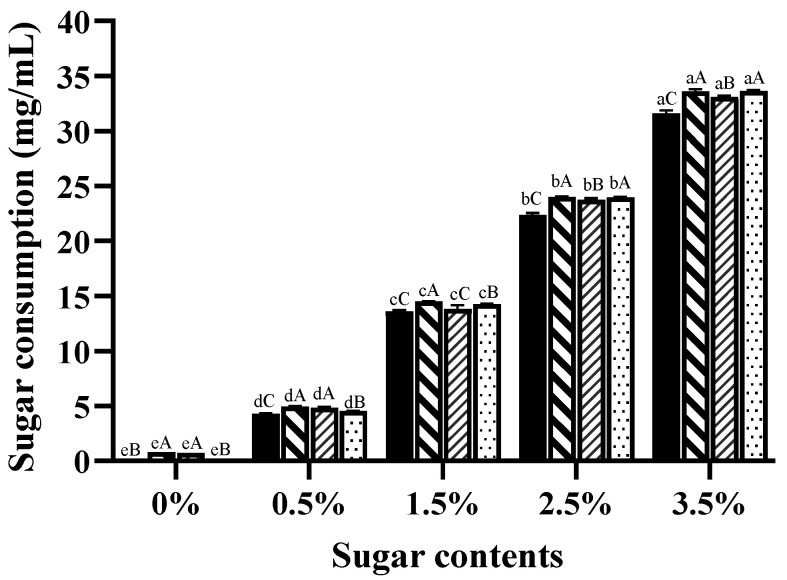
Reducing sugar consumption (mg/mL) in chicken carcass hydrolysates with different added sugars (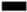 Xylose, 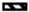 Arabinose, 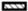 Glucose, 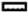 Fructose) after heat treatment at 100 °C for 1 h. ^a,b,c,d,e^ values between the different dosages on the same sugar followed by the same letters are not significantly different (*p* > 0.05). ^A,B,C^ values within the same dosages on different types of sugars followed by the same letters are not significantly different (*p* > 0.05).

The four sugars were barely detectable in both the original chicken carcass hydrolysate (unheated control) and the heated control (xylose: 0 mg/mL; arabinose: 0.8 mg/mL; glucose: 0.72 mg/mL; fructose: 0 mg/mL). Thus, reducing sugars were added to enable the Maillard reaction. All four reducing sugars were consumed in variable amounts, as shown in Figure 2, and this led to varying levels of sugar reduction. Overall, the more sugar was added, the larger the sugar decreased; the largest sugar reduction was 33.63 mg/mL in the fructose-added sample (Fru-35), followed by 33.59 mg/mL in the arabinose-added sample (Ara-35). Fragmentation of reducing sugars in the Maillard reaction could produce numerous substances including volatile flavour compounds (e.g., furans), melanoidins, and Maillard reaction intermediates (MRIs) [33].

### 3.4. Free Amino Acids

The total amount of free amino acids (FAAs) was 446.84 mg/10 mL in the heated control. After heat treatment, there were varying degrees of decline in the total FAAs as the dosage of reducing sugars increased (Figure S2). In general, fructose addition caused the most reduction in FAAs (189.07 mg/10 mL in sample Fru-35), followed by 180.30 mg/10 mL in sample Ara-35, likely due to the higher reactivity of these sugars as mentioned earlier. During the early stage of the Maillard reaction, the terminal α-amino groups of peptides and ε-NH_2_ groups of amino acids interact with the carbonyl group of reducing sugars in the reaction mixture. To compare the sugar reactivity in the MR, another measure that is employed is the loss of accessible primary amino groups. Similar to hexose, fructose should have a lower reduction in amino acids than pentoses; on the other hand, as mentioned in the introduction, fructose tended to be more reactive than glucose, which was in line with other studies [19]. Past research has shown conflicting results on the reactivity of fructose and glucose, with fructose sometimes exhibiting a higher reactivity based on the reaction conditions. This finding may also have been caused by the thermal treatment conditions; it has been demonstrated that conditions such as pH, heating duration, temperature, and moisture/a_w_ factors have a significant impact on the decrease in amino acids [19,28,34]. This study may be notable for its attempt to preserve the highest concentration of taste-active amino acids, while simultaneously producing a high level of aroma flavour components.

**Figure 3:**
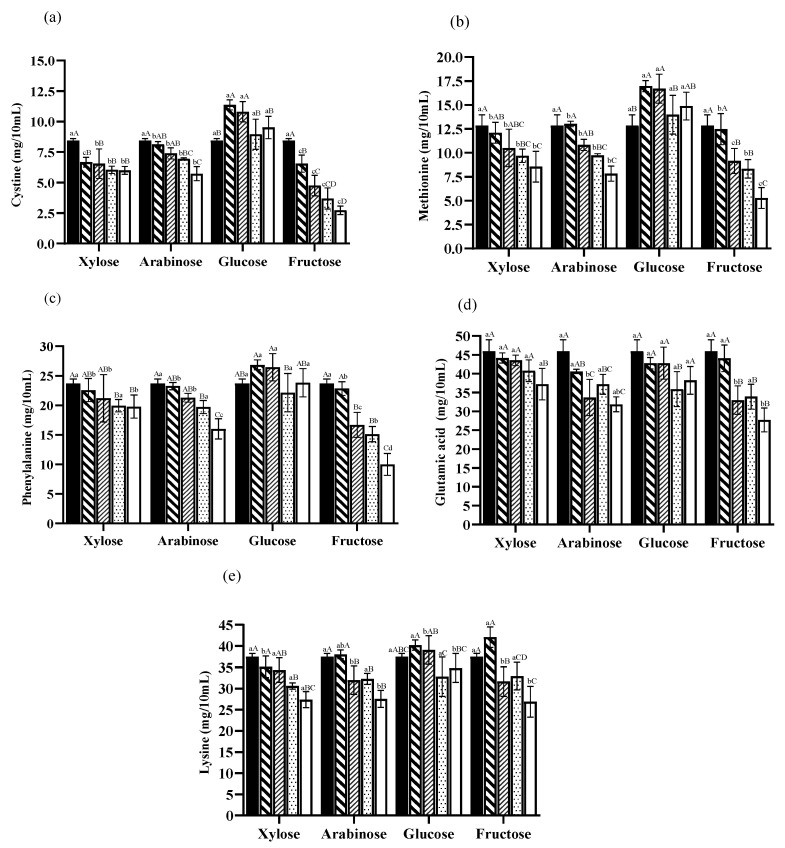
Changes in selected amino acids: cystine (**a**), methionine (**b**), phenylalanine (**c**), glutamic acid (**d**), and lysine (**e**) in heat-treated samples added with different sugars at different dosages (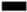 0%, 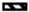 0.5%, 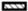 1.5%, 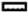 2.5%, 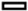 3.5%) after heat treatment at 100 °C for 1 h. ^a,b,c,d^ values within the same dosage on different types of sugars followed by the same letters are not significantly different (*p* > 0.05). ^A,B,C,D^ values between the different sugar dosages on the same sugar followed by the same letters are not significantly different (*p* > 0.05).

To create a meat-based liquid seasoning with desirable aromas such as savoury and meaty notes, it would be necessary to monitor the changes in sulphur-containing amino acids such as cysteine (Cyst), cystine ((Cys)_2_), and methionine (Met) [33]. As shown in Figure 3a, there exists a dose-dependent relationship between cystine reduction and the dosage of arabinose and fructose; the lowest residual (Cys)_2_ amount was 2.73 mg/10 mL in sample Fru-35. Nevertheless, the cystine content in sample Xyl-05 decreased from 8.44 mg/10 mL to 6.67 mg/10 mL, which was statistically significant (*p* < 0.05), but more significant reduction was not achieved if more xylose was added (*p* > 0.05). Interestingly, there was no clear relationship between glucose dosage and cystine reduction. As shown in Figure 3b, in the hexose-added group, the lowest amount of methionine (Met) was found in sample Fru-35 at 5.28 mg/10 mL with an evident dose-dependent relationship between fructose and Met decrease, which was not the case for glucose. Similar to fructose, Met consumption was proportional to the dosage of pentoses. 

In addition, the change in phenylalanine (Phe) was investigated (Figure 3c). There was a general trend of decreasing Phe with increasing dosage of Ara, Xyl, and especially Fru, but not in the case of glucose in which Phe fluctuated. A previous investigation demonstrated that Xyl-Phe had the capability to form an intermediate of the Maillard reaction with a molecular weight of 297 [35]. Through subsequent processes such as dehydration, decarboxylation, and the removal of the furan ring, Xyl-Phe can undergo degradation, ultimately resulting in the formation of benzaldehyde and benzeneacetaldehyde, which could frequently be encountered as flavour components that enhance the overall flavour profile while simultaneously diminishing the perception of bitterness [12,35]. Interestingly, very little recent Ara-Phe related research has been reported. As for the Fru-Phe system, previous research in a model system showed that furfural was the only furan derivative detected, and the formation of phenylacetaldehyde can be due to the Strecker breakdown of phenylalanine [36].

The content of glutamic acid (Glu) decreased consistently but insignificantly in the xylose group (*p* > 0.05) except at 3.5% (*p* < 0.05) (Figure 3d). On the contrary, Glu in sample Fru-35 reduced markedly at 27.72 mg/10 mL (Figure 3d). Significant reductions in Glu mainly occurred at higher dosages of reducing sugars. Lysine (Lys) also plays an important role in MR, including the antioxidation activity and browning degree [37]. Figure 3e shows the changes in Lys. In general, there was no linear relationship between the two hexoses dosage and the Lys content. Furthermore, among the xylose-added samples, the Lys content decreased steadily from 37.47 mg/10 mL to 27.37 mg/10 mL. Furthermore, Fru-35 sample had the lowest Lys content of 26.89 mg/10 mL.

### 3.5. Volatile Compounds

**Figure 4:**
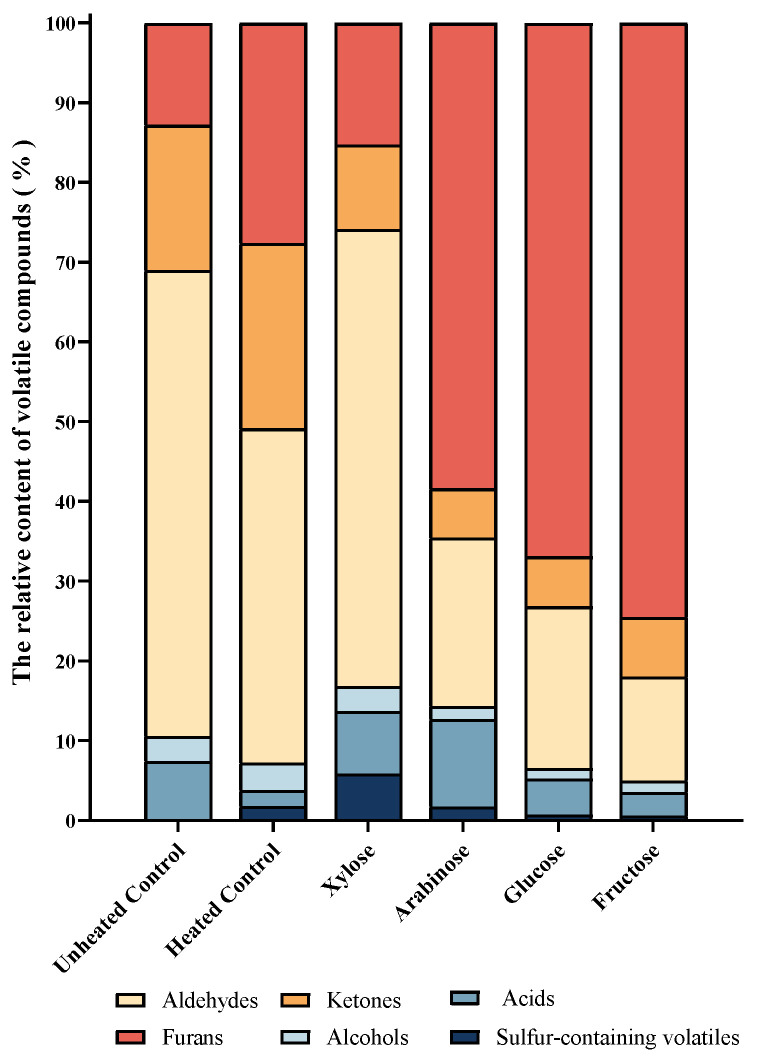
The relative peak areas (RPAs, %) of six groups of volatiles in the total volatile compounds with different sugars added at 2.5% after heat treatment at 100 °C for 1 h.

The volatile compounds are grouped into six groups: acids, alcohols, aldehydes, ketones, furans, and sulphur-containing volatiles. Figure 4 presents the relative peak areas (RPAs, %, Table S1) of each group in the total volatile compounds among the heat-treated samples added with reducing sugars at 2.5%. In general, furans were the dominant compounds in arabinose, glucose, and fructose-added samples (58.39%, 66.90%, 74.48% RPA), while aldehydes had the highest number (nine types) in the xylose-added sample, accounting for 57.31%. Interestingly, the highest number of sulphur-containing volatiles (four types) was found in the xylose-added sample, accounting for 5.88%, followed by arabinose, whereas only one sulphur-containing volatile (2-pentyl-thiophene, accounting for 0.62%) was found in the fructose-added sample, despite it consuming a considerable amount of sulphur-containing amino acids. Fructose addition at high concentration (3.5%) consumed the highest number of amino acids but produced the lowest level of sulphur-containing volatile flavour compounds.

**Figure 5:**
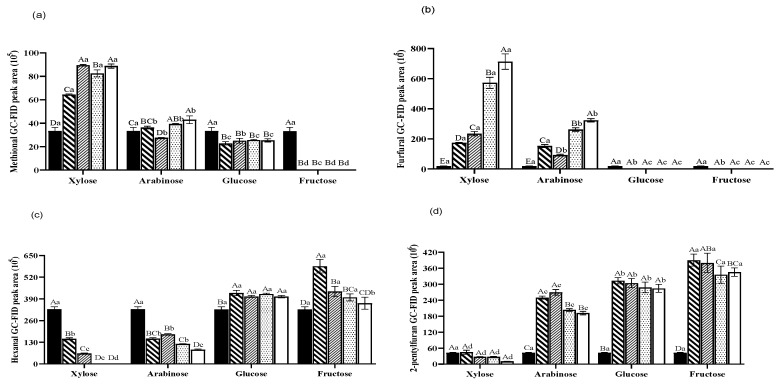
Changes in GC-MS/FID peak areas of methional (**a**) furfural (**b**), hexanal (**c**), and 2-pentylfuran (**d**) in heat-treated samples added with sugars (xylose, arabinose, glucose, and fructose) (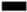 0%, 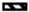 0.5%, 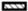 1.5%, 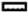 2.5%, 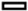 3.5%). ^a,b,c,d^ values within the same dosage on different types of sugars followed by the same letters are not significantly different (*p* > 0.05). ^A,B,C,D^ values between the different dosages on the same sugar followed by the same letters are not significantly different (*p* > 0.05).

Farmer et al. (1994) found that Cyst breakdown during heat treatment would be most desirable since it was anticipated that Cyst would combine with xylose to form meat-like flavour compounds, such as 2-methyl-3-furanthiol (MFT) and 2-furfurylthiol (FFT) [38]. However, the chicken carcass hydrolysate used in our study contained little amount of Cyst, which might explain the lack of MFT and FFT after heat treatment. The highest methional amount was found in sample Xyl-35 (peak area of 89.45 × 10^5^), accounting for 4.09%, which was nearly 2.7 times of the methional content in the control (peak area of 33.22 × 10^5^). Only xylose could boost the generation of methional, and there was no direct relationship between other sugars added and the methional formation (Figure 5a). Previous study showed that Phe and xylose could generate benzeneacetaldehyde after heating at 80 °C for 70 min [35], which was consistent with the result of this study (Table S1).

The change in furfural was obvious (Figure 5b) in pentose-added samples, and the amount of furfural increased significantly with rising xylose and arabinose dosage, except that the furfural content reduced suddenly from a peak area of 169.63× 10^5^ in sample Ara-05 to 92.32 × 10^5^ in sample Ara-15. On the other hand and as expected, the furfural contents of hexoses-added samples were below the detectable level because pentoses are supposed to be dehydrated into furfural, whereas hexoses to hydroxymethylfurfural (HMF) [39,40]. HMF imparts sensory properties (colour, taste, and aroma); however, HMF was not detected in both glucose- and fructose-added samples, which might be caused by the lower heating temperature (100 °C) or higher initial pH value (6.62) and/or the detection method. Furfural is an intermediate formed in the MR in the presence of a pentose; it imparts a robust, spicy, and burned flavour [40,41]. During the cooking of meat, furfural often interacts with hydrogen sulphide, which is produced from the breakdown of Cyst and/other sources, to generate 2-furanmethanethiol, which imparts a strong and distant “roasted meat” aroma to the product [42]. However, 2-furanmethanethiol was not found in this investigation, which may be the result of the low concentration of Cyst in the chicken carcass hydrolysate [17].

There were two opposite trends of hexanal changes between pentoses and hexoses, i.e., with different amounts of added pentoses (Figure 5c), the hexanal level decreased to varying levels, with peak areas of 62.47 × 10^6^ and 177.61 × 10^6^, accounting for 2.91% and 3.81% RPA, respectively. When the amount of xylose exceeded 2.5%, the hexanal content in the heat treatment sample was below the detected level. Contrary to the pentoses, the hexanal levels in hexose-added samples increased first and then were relatively stable in the glucose-added samples, but decreased in the fructose-added samples, with the highest hexanal peak area of 585.78 × 10^6^ in sample Fru-05, accounting for 8.93% RPA. Hexanal is an aliphatic aldehyde that has a grassy odour when present in untreated meat hydrolysate [43]. The high level of hexanal found in different meat products such as cooked poultry meat can be a marker to distinguish the type of meat [44]. These oxidised lipids played a crucial part in the Maillard process and the formation of volatile compounds such as pyrazines, thiazoles, and thiols [42,45]. According to a previous investigation [42], the reduction in hexanal could also indicate its interference in the interaction between cysteine and reducing sugars (Figure 3a and Figure 5c).

Overall, there were dramatic increases in 2-pentylfuran in sugar-added samples, except xylose (Figure 5d). In contrast, the xylose-added sample had the highest level of furfural (Figure 5b), which was predominant in the compounds generated by the heat treatment. Furfural could easily participate in further Strecker degradation and condensations with amino acids, leading to the formation of melanoidins that may have strong antioxidative properties, whereas 2-pentylfuran is often produced by the oxidation of linoleic acid and imparts buttery, green, and beany characteristics to foodstuffs when present at 1–10 ppm [46,47]. Xu et al. (2011) also revealed that MRPs affected the production of lipid oxidation products, which means 2-pentylfuran could be a marker for the lipid oxidation degree [42]. Although 2-pentylfuran generated after heat treatment does not directly provide a desired “meaty” aroma, it has been identified as a contributor to the overall broiled or roasted-meat aroma in certain sauce products [48]. 

## 4. Conclusions

The addition of two pentoses (xylose and arabinose) and two hexoses (glucose and fructose) led to different degrees of pH decrease and browning in the chicken carcass hydrolysate after heat treatment. Variable quantities of added sugars were used. There was a dose-dependent relationship between each sugar dosage and the colour change (∆E values). The volatile flavour compound profiles were affected by the sugar type significantly. Pentoses addition at the same dosage could generate more sulphur-containing volatile compounds than hexoses, such as methional, 2-[(methylthio) methyl] furan and dimethyl disulphide. However, typical meat-like volatile flavour compounds such as 2-methyl-3-furanthiol (MFT) were not found due to the deficiency of cysteine.

## Figures and Tables

**Table 1:** Heat treatment conditions for chicken carcass hydrolysates.

Sample Name ^1^	Sugar Added (g/100 g)				Temperature (°C)	Time (min)
	Xylose	Arabinose	Glucose	Fructose		
Unheated control ^2,3^	0	0	0	0	-	-
Heated Control	0	0	0	0	100	60
Xyl-05	0.5	0	0	0	100	60
Xyl-15	1.5	0	0	0	100	60
Xyl-25	2.5	0	0	0	100	60
Xyl-35	3.5	0	0	0	100	60
Ara-05	0	0.5	0	0	100	60
Ara-15	0	1.5	0	0	100	60
Ara-25	0	2.5	0	0	100	60
Ara-35	0	3.5	0	0	100	60
Glu-05	0	0	0.5	0	100	60
Glu-15	0	0	1.5	0	100	60
Glu-25	0	0	2.5	0	100	60
Glu-35	0	0	3.5	0	100	60
Fru-05	0	0	0	0.5	100	60
Fru-15	0	0	0	1.5	100	60
Fru-25	0	0	0	2.5	100	60
Fru-35	0	0	0	3.5	100	60

“-”: not applied. ^1^ there were four replications of each treatment for data acquisitions (*n* = 4). ^2^ unheated control was prepared and incubated at room temperature (25 °C) for 1 h. ^3^ the unadjusted pH was 6.62 ± 0.05.

**Table 2:** Colour changes in control and heat-treated chicken carcass hydrolysates.

Sample Name	Colour			
	L*	a*	b*	∆E ^a^
Unheated control	83.77 ± 1.33	1.51 ± 0.32	42.23 ± 0.92	-
Heated control	80.55 ± 0.99	2.96 ± 0.44	49.22 ± 0.46	10.57 ± 0.82
Xyl-05	62.11 ± 0. 20 Ab	30.40 ± 0. 16 Cc	84.22 ± 0. 13 Aa	55.38 ± 0.21 Da
Xyl-15	28.64 ± 0. 52 Bc	45.81 ± 0. 18 Ab	49.13 ± 0. 95 Ba	71.06 ± 0.21 Ca
Xyl-25	13.96 ± 0. 19 Cc	38.49 ± 0. 21 Ba	23.70 ± 0. 40 Ca	81.15 ± 0.17 Ba
Xyl-35	6.29 ± 0. 09 Dc	30.18 ± 0. 18 Ca	10.22 ± 0. 14 Db	88.59 ± 0.07 Aa
Ara-05	59.38 ± 0. 32 Ab	22.01 ± 0. 31 Cc	71.65 ± 0. 34 Aa	43.37 ± 0.49 Db
Ara-15	34.81 ± 0. 43 Bc	41.19 ± 0. 37 Ab	59.27 ± 0. 66 Ba	65.29 ± 0.26 Cb
Ara-25	21.74 ± 0. 77 Cc	41.43 ± 0. 40 Aa	36.96 ± 1. 40 Cb	73.96 ± 0.55 Bb
Ara-35	14.13 ± 0. 42 Dc	37.59 ± 0. 40 Ba	23.76 ± 0. 74 Db	80.58 ± 0.35 Ab
Glu-05	76.92 ± 0. 13 Aa	5.10 ± 0. 05 Dc	53.33 ± 0. 18 Db	13.53 ± 0.12 Dc
Glu-15	75.16 ± 0. 14 Ba	8.01 ± 0. 14 Cc	59.64 ± 0. 34 Cb	20.35 ± 0.28 Cc
Glu-25	72.32 ± 0. 30 Ca	12.08 ± 0. 14 Bc	66.90 ± 0. 09 Bb	29.18 ± 0.17 Bc
Glu-35	69.93 ± 0. 22 Db	15.83 ± 0. 22 Ac	73.15 ± 0. 78 Aa	36.78 ± 0.74 Ac
Fru-05	78.80 ± 0. 49 Aa	5.49 ± 0. 10 Bc	56.62 ± 0. 09 Cb	15.74 ± 0.12 Cd
Fru-15	79.51 ± 0. 13 Aa	5.90 ± 0. 87 Bc	58.26 ± 2. 31 Cb	17.16 ± 2.41 Cd
Fru-25	78.85 ± 0. 21 Aa	7.75 ± 0. 04 Ac	62.43 ± 0. 13 Bb	21.70 ± 0.11 Bd
Fru-35	78.00 ± 0. 53 Aa	9.27 ± 0. 25 Ac	65.28 ± 0. 09 Ab	25.00 ± 0.27 Ad
Test of significance between effects, P				
Main effect				
Sugar type	***	***	***	***
Sugar dosage	***	***	***	***
Sugar type × dosage	***	***	***	***

^a^ Different capital letters of ΔE indicate significant differences among the groups with the same sugar at different dosages, while lowercase letters indicate significant differences among the groups with different sugars at the same dosage. All values are the mean ± standard deviation with three replications of each sample treatment (*n* = 3). ^a,b,c,d^ values within the same sugar dosage on different types of sugars followed by the same letters are not significantly different (*p* > 0.05). ^A,B,C,D^ values between the different sugar dosages on the same sugar followed by the same letters are not significantly different (*p* > 0.05). ***: Significant differences (*p* < 0.001).

## Data Availability

The original contributions presented in the study are included in the article/Supplementary Materials, further inquiries can be directed to the corresponding author.

## Supplementary Materials

The following supporting information can be downloaded at: https://www.mdpi.com/article/10.3390/foods13070991/s1, Figure S1: Colour changes in unheated control and heat-treated chicken carcass hydrolysates; Figure S2: Change in total free amino acids in heat-treated samples added with different sugars at different dosages ( 0%,  0.5%,  1.5%,  2.5%,  3.5%) after heat treatment at 100 °C for 1 hr. ^A,B,C,D^ values within the same sugar dosage on different sugars followed by the same letters are not significantly different (*p* > 0.05). ^a,b,c,d^ values between the different sugar dosages on the same sugar added followed by the same letters are not significantly different (*p* > 0.05). Table S1: Average FID-GC peak areas (×106) and relative peak areas (RPAs, %) of volatiles in the blank, control, and heated chicken carcass hydrolysates with different added sugars (2.5%).
