# Supplementary material for: Effects of Reducing Sugars on Colour, Amino Acids, and Volatile Flavour Compounds in Thermally Treated Minced Chicken Carcass Hydrolysate
Source: Foods. 2024 Mar 24;13(7):991. doi: 10.3390/foods13070991 (PMC11011280; doi:10.3390/foods13070991)
Supplement: Supplementary file 1 [file foods-13-00991-s001.zip › foods-2927528-supplementary.pdf]

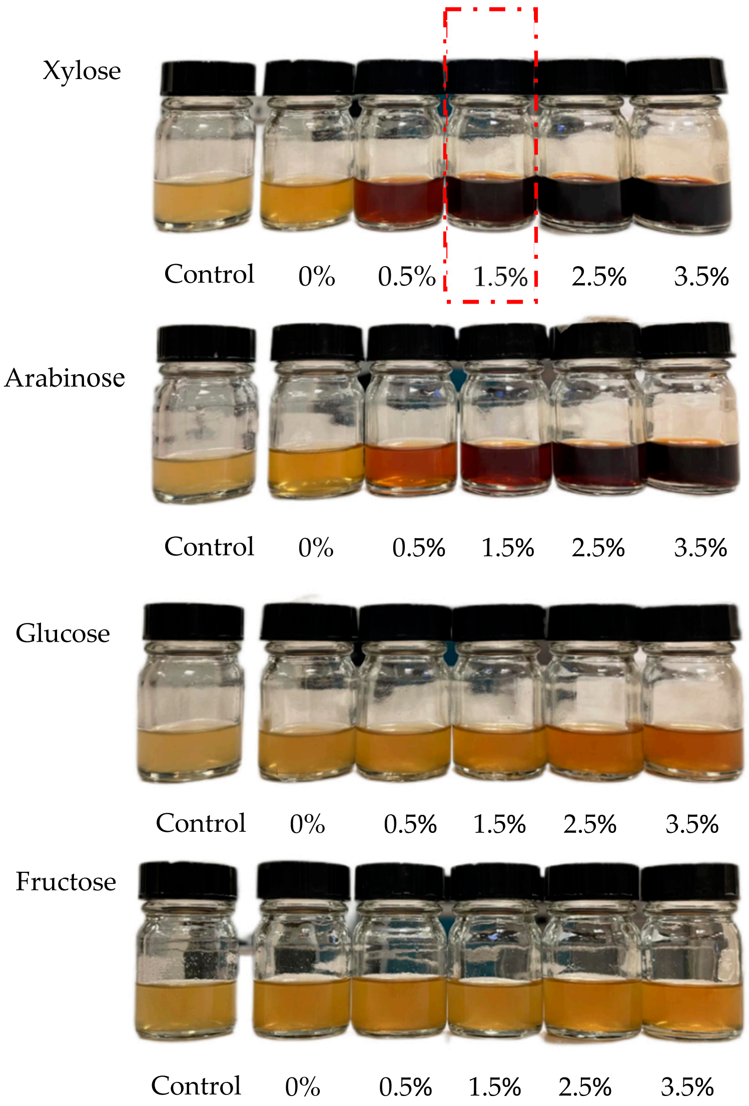

Figure S1. Colour changes of unheated control and heat-treated chicken carcass hydrolysates.

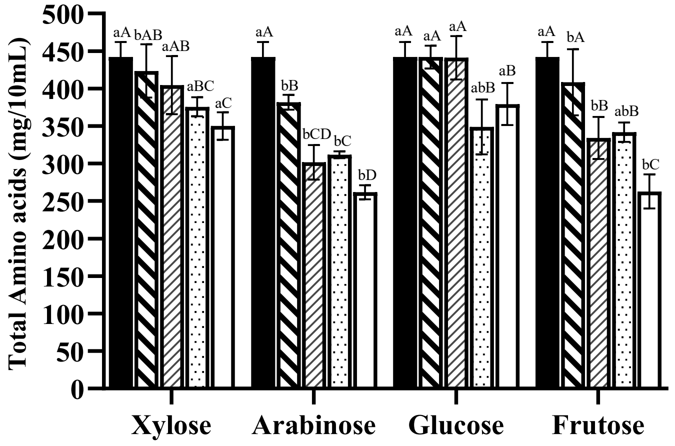

Figure S2. Change in total free amino acids in heat-treated samples added with different sugars at different dosages (0%, 0.5%, 1.5%, 2.5%, 3.5%) after heat treatment at 100 °C for 1 hr. ^{A,B,C,D} values within the same sugar dosage on different sugars followed by the same letters are not significantly different ($p > 0.05$). ^{a,b,c,d} values between the different sugar dosages on the same sugar added followed by the same letters are not significantly different ($p > 0.05$).

Table S1. Average FID-GC peak areas (×10[6]) and relative peak areas (RPA, %) of volatiles in the blank, control and heated chicken carcass hydrolysates with different added sugars (2.5%).

	Name	LRI Exp.	Unheated Control	Heated Control	Xylose	Arabinose	Glucose	Fructose
	Sulfur-containing volatiles							
1	Methional	1083	-	3.32 ± 0.31 B	8.25 ± 0.31 A	3.93 ± 0.04 B	2.56 ± 0.05 B	-
2	2-[(methylthio)methyl]-furan	1067	-	-	2.48 ± 0.09	2.58 ± 0.09	-	-
3	Dimethyl disulfide	1072	-	-	5.14 ± 0.24	-	-	-
4	Dimethyl trisulfide	1030	-	-	2.74 ± 0.48	-	-	-
5	3,4-Dimethyl-thiophene	1893	-	-	-	0.81 ± 0.07	-	-
6	4-Methoxy-benzenethiol	1050	-	-	-	-	1.23 ± 0.03	-
7	2-Pentyl-thiophene	1075	-	-	-	-	-	3.54 ± 0.14
	Subtotal		-	3.32 ± 0.31 C	18.61 ± 1.12 A	7.32 ± 0.20 B	3.79 ± 0.08 C	3.54 ± 0.14 C
	RPA (%)		-	1.65%	5.76%	1.84%	0.76%	0.62%
	Non-sulfur volatiles							
	Acids							
1	Acetic acid	1074	-	-	5.56 ± 0.41	8.11 ± 0.38	-	0.80 ± 0.09
3	Hexanoic acid	1072	-	-	-	3.79 ± 0.04	2.68 ± 0.04	3.05 ± 0.11
4	Heptanoic acid	1019	-	-	-	2.97 ± 0.28	2.34 ± 0.19	0.96 ± 0.08
5	Octanoic acid	1066	5.25 ± 0.64 E	-	9.40 ± 0.54 C	17.08 ± 0.93 A	13.05 ± 0.73 B	8.48 ± 0.52 D
6	Nonanoic acid	1010	2.67 ± 0.11 C	2.35 ± 0.16 C	3.37 ± 0.31 B	5.12 ± 0.24 A	3.18 ± 0.40 B	3.46 ± 0.31 B
7	n-Decanoic acid	1057	-	-	2.04 ± 0.35	1.57 ± 0.10	-	-
8	Benzoic acid	1050	-	1.31 ± 0.22 A	0.89 ± 0.06 A	1.17 ± 0.11 A	0.93 ± 0.06 A	-
9	Dodecanoic acid	1067	-	-	3.51 ± 0.42	5.31 ± 0.37	-	-
	Subtotal		7.92 ± 0.75 F	15.06 ± 1.78 E	24.77 ± 2.09 B	45.12 ± 2.45 A	22.18 ± 1.42 C	16.75 ± 1.11 D
	RPA (%)		3.59%	7.46%	8.70%	9.05%	4.34%	2.92%
	Alcohols							
1	2-Furanmethanol	1098	-	5.30 ± 0.59 B	7.54 ± 0.05 A	5.47 ± 0.09 B	4.92 ± 0.20 B	5.55 ± 0.47 B
2	1-Pentanol	1040	3.31 ± 0.24	-	-	-	-	-
3	3,5-Octadien-2-ol	1047	-	-	-	-	-	1.31 ± 0.10
4	2,4-Di-tert-butylphenol	1073	-	1.08 ± 0.06 A	2.34 ± 0.25 A	1.31 ± 0.11 A	1.58 ± 0.19 A	1.62 ± 0.15 A
5	***Subtotal***		3.31 ± 0.24 D	6.38 ± 0.65 C	9.88 ± 0.30 A	6.78 ± 0.20 C	6.50 ± 0.39 C	8.48 ± 0.72 B

6	*RPA (%)*		3.25%	3.16%	3.06%	0.33%	1.31%	1.48%
	Aldehydes							
1	2-Methyl-butanal	<900	-	-	79.10 ± 1.43	-	-	-
2	3-Methyl-butanal	<900	-	3.73 ± 0.52	-	-	-	-
3	Pentanal	<900	4.52 ± 0.05 B	3.15 ± 0.43 C	-	-	5.30 ± 0.18 B	5.39 ± 0.40 A
4	Hexanal	1080	45.04 ± 0.56 A	32.77 ± 1.55 D	-	11.95 ± 0.25 E	41.92 ± 0.48 B	39.89 ± 2.08 C
5	(*E*, *E*)-2,4-Decadienal	1088	-	1.72 ± 0.19 B	2.12 ± 0.03 B	9.89 ± 0.44 A	10.80 ± 0.90 A	-
6	Furfural	1089	-	1.89 ± 0.06 C	57.22 ± 3.72 A	26.19 ± 1.21 B	-	-
8	Octanal	1061	2.24 ± 0.20	-	-	-	-	5.36 ± 0.29
9	(*E*)-2-Heptenal	1094	2.93 ± 0.15	-	-	-	-	-
10	Nonanal	1043	0.21 ± 0.03 C	4.22 ± 0.55 B	4.21 ± 0.45 B	6.36 ± 0.29 B	9.86 ± 0.21 A	0.93 ± 0.06 C
11	(*E*)-2-Octenal	1065	-	-	-	-	2.25 ± 0.06	-
12	Benzaldehyde	1027	7.07 ± 1.04 E	23.97 ± 3.81 A	18.08 ± 0.59 B	15.50 ± 0.30 C	15.77 ± 0.52 C	11.62 ± 0.37 D
13	Benzene acetaldehyde	1089	-	2.49 ± 0.16 C	13.63 ± 0.61 A	10.11 ± 0.55 B	3.29 ± 0.20 C	-
14	4-Ethyl-benzaldehyde	1023	-	-	-	2.04 ± 0.10	-	3.12 ± 0.28
15	3-Ethyl-benzaldehyde	1024	-	-	-	-	4.73 ± 0.18	-
16	2,5-Furandicarboxaldehyde	1076	-	-	1.56 ± 0.04			-
17	3,5-Dimethyl-benzaldehyde	1080	-	-		2.51 ± 0.20	4.50 ± 0.44	-
18	3,4-Dimethyl-benzaldehyde	1080	-	3.53 ± 0.38	2.15 ± 0.27	-	-	-
19	2,4-Dimethyl-benzaldehyde	1078	-	-	-	-	-	3.11 ± 0.16
20	5-Methyl-2-phenyl-2-hexenal	1085	-	-	3.32 ± 0.40	2.61 ± 0.10	-	-
21	2-Butyl-2-octenal	1093	-	-	-	-	2.39 ± 0.12	2.54 ± 0.09
22	2-Butylhept-2-enal	1045	-	-	-	-	-	0.90 ± 0.06
23	(*E*)-2-Tridecenal	1063	-	-	-	-	-	1.58 ± 0.18
	Subtotal		62.01 ± 2.03	77.47 ± 7.65	181.39 ± 7.54	87.16 ± 3.44	100.81 ± 3.29	74.44 ± 3.97
	RPA (%)		60.88%	38.39%	56.10%	21.88%	20.31%	12.99%
	Ketones							
1	4-Methyl-3-penten-2-one	1031	7.73 ± 0.50 B	30.57 ± 0.77 A	9.63 ± 0.13 B	5.60 ± 0.10 D	-	7.59 ± 0.51 C
2	3-Hexen-2-one	1031	-	-	-	-	6.93 ± 0.26	-
3	2-Heptanone	1071	4.80 ± 0.16 C	8.25 ± 1.19 B	3.24 ± 0.01 C	5.62 ± 0.24 C	9.78 ± 0.42 B	18.38 ± 1.57 A
4	2-Methyl-3-Octanone	1053	-	-	2.39 ± 0.03	-	-	-
5	2-Octanone	1057	0.31 ± 0.04 B	1.31 ± 0.05 B	2.38 ± 0.07 B	3.37 ± 0.18 B	1.58 ± 0.05 B	5.10 ± 0.44 A
6	3-Ethylcyclopentanone	1098	2.85 ± 0.30	-	-	-	-	-

7	3-Octen-2-one	1060	0.59 ± 0.08 A	0.70 ± 0.07 A	0.59 ± 0.07 A	1.21 ± 0.03 A	1.35 ± 0.07 A	-
9	2,3-Octanedione	1088	3.06 ± 0.28	-	-	-	-	-
10	2-Nonanone	1034	-	1.01 ± 0.11 A	1.42 ± 0.14 A	1.50 ± 0.04 A	1.57 ± 0.08 A	3.48 ± 0.07 A
11	1-(2-Furanyl)-1-propanone	1053	-	-	0.82 ± 0.07	-	-	-
12	2-Decanone	1098	-	1.14 ± 0.15 D	1.58 ± 0.07 C	2.96 ± 0.16 B	2.95 ± 0.04 B	8.25 ± 0.35 A
13	1-(2-furanylcyclopropyl)-ethenone	1002	-	-	-	-	2.52 ± 0.06	-
14	trans-3-Nonen-2-one	1012	-	-	-	3.76 ± 0.37	2.47 ± 0.28	-
15	2-pentyl-2-cyclopenten-1-one	1039	-	-	-	-	2.07 ± 0.03	-
16	2 (5 *H*)-Furanone	1052	-	-	1.56 ± 0.18	1.42 ± 0.10	-	-
17	4-Hydroxy-5-methyl-3(2*H*)-furanone	1001	-	-	9.87 ± 0.76	-	-	-
	Subtotal		19.34 ± 1.36	48.51 ± 2.94	33.48 ± 1.53	25.44 ± 1.22	31.22 ± 1.29	43.67 ± 2.99
	RPA (%)		18.99%	24.04%	10.36%	6.39%	6.29%	7.62%
	Furans							
1	2-Ethylfuran	<900	-	5.07 ± 0.33 D	-	6.55 ± 0.10 C	8.25 ± 1.15 B	15.43 ± 1.68 A
2	2-Propyl-Furan	1030	-	-	-	-	-	4.47 ± 0.34
3	2-Methylfuran	<900	-	-	10.39 ± 0.12	1.26 ± 0.12	-	-
4	2-n-Butylfuran	1016	1.16 ± 0.14	-	-	3.30 ± 0.15	5.33 ± 0.48	6.86 ± 0.31
5	2-Pentylfuran	1007	11.74 ± 0.90 F	42.83 ± 1.38 E	26.96 ± 1.98 D	203.25 ± 5.02 C	287.94 ± 19.99 B	345.52 ± 16.01 A
7	2-n-Octylfuran	1019	-	0.69 ± 0.06 A	0.72 ± 0.06 A	1.56 ± 0.09 A	1.46 ± 0.13 A	1.07 ± 0.05 A
8	cis-2-(2-Pentenyl) furan	1067	0.64 ± 0.05 E	2.48 ± 0.22 D	2.87 ± 0.29 D	11.93 ± 2.06 C	19.05 ± 2.06 B	28.07 ± 2.36 A
9	2-Hexylfuran	1087	-	-	-	-	-	13.09 ± 1.18
10	(*E*)-2-(1-pentenyl)-furan	1017	-	-	-	-	-	1.62 ± 0.08
11	2-n-Heptylfuran	1058	-	-	0.62 ± 0.07 D	5.07 ± 0.23 C	8.93 ± 0.30 B	9.04 ± 0.73 A
12	3-Phenylfuran	1090	-	-	6.81 ± 0.28 B	8.19 ± 0.57 A	1.53 ± 0.17 C	0.94 ± 0.09 C
	Subtotal		13.54 ± 1.09	51.07 ± 1.99	51.83 ± 2.86	241.11 ± 8.34	332.49 ± 24.28	426.11 ± 22.83
	RPA (%)		13.29%	25.31%	16.03%	60.52%	66.99%	73.37%
	Total		106.12 ± 4.72	201.8 ± 15.86	323.32 ± 15.12	398.40 ± 15.49	496.34 ± 30.60	572.99 ± 31.76

LRI: linear retention index. -: Compounds not detected. Data are the mean ± SD (n = 3). A–F Values in the same row with different letters were significantly different ($p < 0.05$).
